# Deposition is a phosphorus source for *Fallopia japonica* during early-stage primary succession

**DOI:** 10.1038/s41598-023-42935-z

**Published:** 2023-09-25

**Authors:** Sae Katayama, Koichiro Sawakami, Masaki Tateno

**Affiliations:** Nikko Botanical Garden, Graduate School of Science, University of Tokyo, Nikko, Tochigi, Japan

**Keywords:** Plant ecology, Element cycles

## Abstract

Phosphorus is a key plant nutrient linked to plant growth during the early stages of primary succession in volcanic soils. Available phosphorus is thought to include soil and atmospheric phosphorus, but it is not well understood. Here, we focused on deposition as a potential phosphorus source. We evaluated the contribution of deposition to phosphorus uptake and growth in *Fallopia japonica*, a key pioneer species of primary succession. When we experimented with growing *F. japonica* under field conditions, *F. japonica* not covered by a roof absorbed more phosphorus than that covered by the roof, suggesting the influence of total (dry + wet) deposition. Furthermore, we tested the effects of deposition by treating *F. japonica* seedlings with wet deposition or distilled water in six volcanic soils. Plants that received the wet deposition treatment exhibited higher phosphorus contents and growth rates than those treated with distilled water. The phosphorus from wet deposition and the phosphorus from soil contributed nearly equally to *F. japonica* development. Our findings suggest that *F. japonica* grows during primary succession and builds up the phosphorus cycle by absorbing a trace amount of phosphorus from deposition and volcanic soils.

## Introduction

Nitrogen and phosphorus are common limiting nutrients for plant growth in natural environments^1^. This is especially notable in volcanic soils during the early stages of primary succession, which are deficient in both nitrogen and phosphorus. *Fallopia japonica* (Houtt.) Ronse Decr. var. *japonica* is an opportunistic pioneering species that establishes itself during the early stages of primary succession and has no innate nitrogen-fixing capacity from the atmosphere^2,3^. In immature volcanic soils, the roots of *F. japonica* seedlings absorb inorganic nitrogen from wet deposition (precipitation) to colonize^3,4^.

Although wet deposition is recognized as the primary source of nitrogen, the source of phosphorus for *F. japonica* during the early stages of primary succession still needs to be elucidated. Litter is considered a main source of phosphorus in volcanic soils^5^, but plant litter is not present on the soil surface during the early stages of primary succession. In these immature soils, phosphorus exists in mineral in the form of calcium phosphate, which gradually dissolves during weathering^6^. However, dissolved phosphorus is strongly adsorbed by aluminum and iron in the volcanic soil, making it insoluble over time^6^. Therefore, volcanic soils cannot supply sufficient phosphorus for plants to grow easily, especially older soils. For these reasons, we investigated the source of phosphorus that *F. japonica* utilizes during the early stages of primary succession in volcanic soils.

Here, we hypothesized two possibilities. One is phosphorus dissolution by acidic deposition. Wet deposition is typically weakly acidic due to acidic pollutants (such as sulfur dioxide and nitrogen oxides) and high inputs of acidic deposition increase the leaching of calcium and other cations from soil^7^. Therefore, we tested the possibility that inorganic phosphorus is produced as a result of cations leaching, and that pioneer plants would utilize this inorganic phosphorus. The second hypothesis is phosphorus supply by deposition. The concentration of inorganic phosphorus (PO_4_-P) in wet deposition measured in Japan is reported to be approximately 0.03 mg/L^8^. Furthermore, atmospheric aerosols have been reported to contain phosphorus, possibly emanating from terrestrial sources^9^. Phosphorus uptake by vegetation is roughly less than 100 mg/m^2^ per year in the early stage of primary succession on Hawaiian volcanic soils (approximately 30–100 mg/m^2^ phosphorus uptake in the area from 700 to 1660 m elevation)^10^. Atmospheric phosphorus deposition in agricultural regions has also been reported to be 30 mg/m^2^ per year^11^, suggesting that phosphorus deposition can be an important phosphorus source during the early stage of primary succession. Long-range transport of dust from Asia, 6000 km away, is an important source of phosphorus in the Hawaiian Islands^12^. However, the effect of deposition on phosphorus uptake and growth in the pioneer species of *F. japonica* has not been studied.

In this study, we investigate the role of deposition as a phosphorus source for *F. japonica*. First, we examine whether the pH of weakly acidic wet deposition promotes the dissolution of phosphorus in young volcanic soils, contributing to phosphorus absorption by *F. japonica* (experiment #1). Next, we evaluate the effect of total (wet + dry) deposition on phosphorus supply by examining phosphorus absorption in *F. japonica* with and without exposure to total deposition (experiment #2). Thirdly, we study the effects of wet deposition and soil age on phosphorus absorption by *F. japonica* by growing plants with wet deposition or distilled water conditions in six different volcanic soils of various ages (experiment #3). Finally, we attempted to determine whether nitrogen or phosphorus is the growth-limiting inorganic nutrient for *F. japonica* in these six volcanic soils containing little nitrogen and phosphorus (experiment #4). This study will provide important experimental evidence for understanding plant phosphorus cycling during the early stage of primary succession in volcanic soil ecosystems.

## Materials and methods

### Volcanic soils

Six types of volcanic soils were prepared (Table 1). Four soils were collected by us in 2020, and the remaining two were obtained commercially. All soils were sieved to obtain particles with a diameter of 6 mm or less for experimental use. The approximate locations of the soils used in this study are summarized in Supplementary Fig. S1.

**Table 1:** Ages and types of the six soils.

Soil name	Soil type	Approximate age (year)	Reference
Sakurajima (SJ)	ash	< 1	
Fuji (FJ)	scoria	300	^16^
Haruna (HR)	pumice	1500	^13^
Nasu (NS)	ash, lapilli composed of clayey matrix	600–2600	^14^
Kanuma (KN)	pumice	44,000	^17^
Kiryu (KR)	welded tuff	60,000,000	^15^

The four soils collected were Sakurajima (SJ), Haruna (HR), Nasu (NS), and Kiryu (KR) soil. Only immature soil was collected using a shovel, excluding fallen leaves, roots, and other large organic matter if any. Soil sampling was conducted on the surface of outcrops where immature soil was present. Fresh volcanic ash (SJ) was collected from a street in Tarumizu City within a few months of the eruption of the Sakurajima volcano (no collection permit was required). HR was a Futatsudake pumice deposited approximately 1500 years ago by the eruption of Mt. Haruna^13^. The HR collection site (36° 28′ 56″ N, 138° 53′ 59″ E) was a forest road managed by Shibukawa City, and we received permission to collect the samples. NS was an eruptive deposit of the Nasu-Chausudake volcano, approximately 600–2600 years in age (1408–1410 & Minenochaya units)^14^. Because the NS site (37° 7′ 23″ N, 139° 58′ 9″ E) was within Nikko National Park, we obtained a collection permission (No. 2006013) from the Nikko National Park Office of the Ministry of the Environment. KR was a Kanayama welded tuff deposited about 60 million years ago^15^. The KR collection site (36° 22′ 38″ N, 139° 19′ 52″ E) was on private land, and permission was obtained from the landowner to collect soil.

The commercial soils were Fuji (FJ) and Kanuma (KN) soils. FJ (Yoshoen Inc.) was a scoria deposited during the 1707 Hoei eruption of Mt. Fuji with an age of approximately 300 years^16^. KN (Akagi Engei Co., Ltd.) was a fine-grained pumice deposited approximately 44,000 years ago by the eruption of Mt. Akagi^17^.

**Figure 1:**
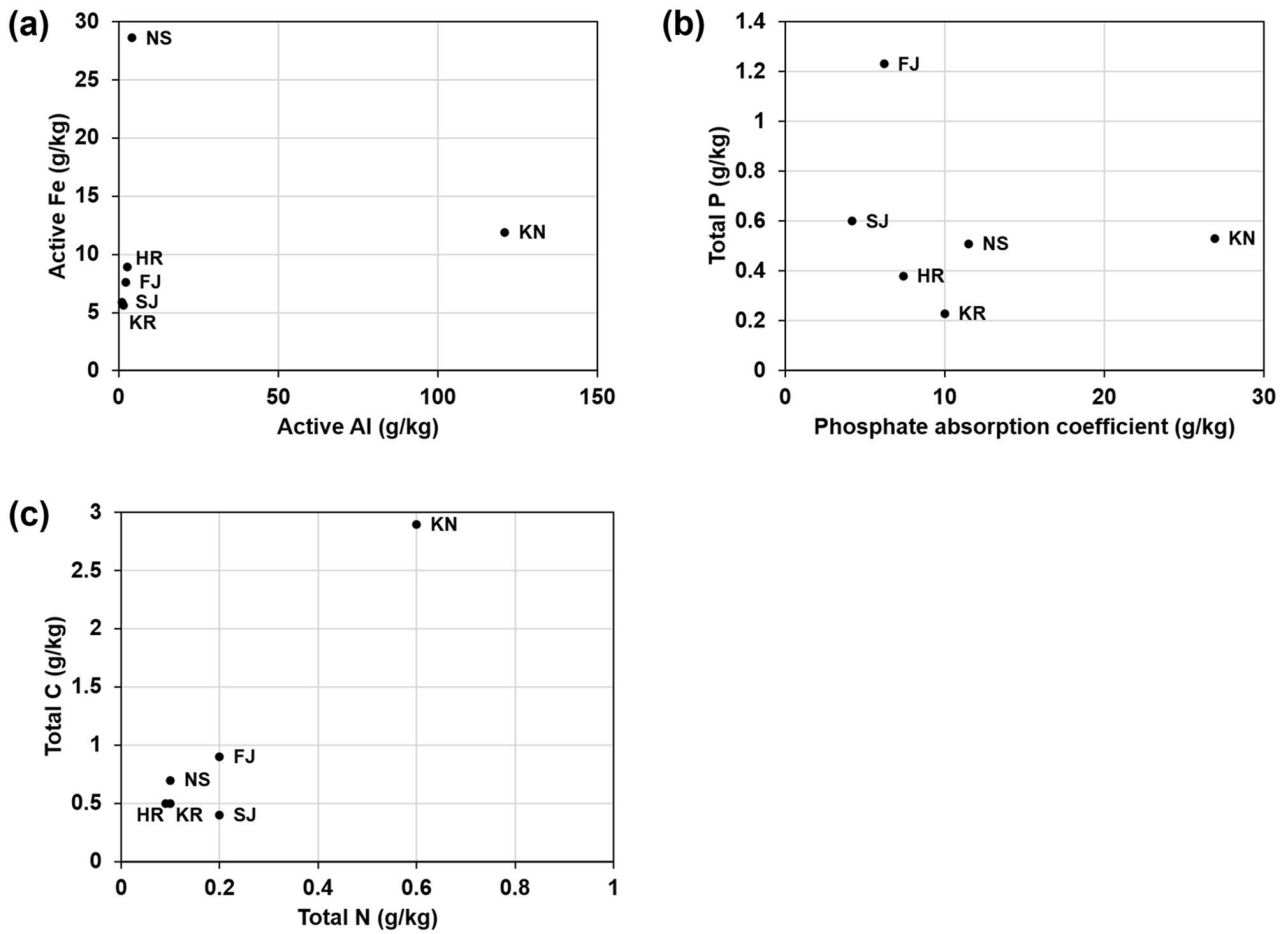
Soil properties. (**a**) Active Al and Fe. (**b**) Total P and phosphorus absorption coefficient. (**c**) Total C and N. The raw data are available in Supplementary Table S1.

Soil analysis for total phosphorus, total nitrogen, total carbon, phosphate absorption coefficient, active aluminum, and active iron was performed by Createrra Inc. (http://www.createrra.co.jp/english/top.html) (Fig. 1). Active aluminum and iron were measured using the acid oxalate extraction method^18^. The phosphate absorption coefficient, an indicator of the magnitude of phosphorus adsorption in soils commonly used in Japan, was used to evaluate the availability of phosphorus in the soil^19^. The higher the phosphate absorption coefficient, the more strongly the soil fixes phosphorus. No trend was found for active Al, active Fe, or phosphate absorption coefficient with soil age (Fig. 1a,b, Table 1). KN had higher active Al and phosphate absorption coefficients than the other soils (Fig. 1a,b) and was comparable to andosol, a mature volcanic soil with high phosphorus adsorption properties^19^. Total phosphorus, total nitrogen, and total carbon contents in the volcanic soils were similar in all soils; however, total phosphorus in FJ was slightly higher than in the other soils (Fig. 1b,c).

### Wet deposition

Although nutrient conditions for wet deposition may vary depending on the collection site, in this study, wet deposition was regularly collected at our research site (NBG), and used for the experiment. Wet deposition was collected in polyethylene tanks in the open space of the NBG (36° 45′ N, 139° 35′ E; 647 m a.s.l.) on rainy days from October 2019 to December 2020 for growth experiment #3. Wet deposition collected in tanks was filtered through a nylon mesh (mesh size approximately 0.3 mm) to remove large debris. The wet deposition was collected when it stopped and stored at room temperature (approx. 5 °C to 25 °C) in tanks for subsequent use.

Wet deposition used for chemical analysis was similarly collected on November 1, 2022, and January 24–25, 2023 (Table 2). It was collected when the wet deposition stopped. Precipitation amount was 4 mm on November 1, 2022, and 5 mm on January 24–25, 2023. Wet deposition was refrigerated immediately after collection. Carbon analysis (total carbon, total organic carbon, and inorganic carbon) was performed using a TOC/TN analyzer (multi N/C 3100, Analytik Jena AG). The analysis of phosphorus (inorganic phosphorus, PO_4_-P), chloride ion, and sulfate ion was analyzed using ion chromatography (Dionex Integrion RFIC, Thermo Fisher Scientific K.K.). Inorganic phosphorus of wet deposition on November 1, 2022 was below the limit of quantitation (< 0.008 mg/L) and could not be detected. Hence, the wet deposition collected on January 24–25, 2023, was concentrated 250-fold in a lyophilizer and the filtrate was used for phosphorus analysis. The phosphorus values in Table 2 were converted back to the original wet deposition concentration. The analysis of nitrogen (inorganic nitrogen, NO_3_-N, NO_2_-N, and NH_4_-N), potassium, calcium, magnesium, and sodium content of the wet deposition on November 1 was conducted by K.S Environmental Research Institute Co., Ltd (https://ks-kankyo.co.jp/). The analysis of nitrogen (inorganic nitrogen, NO_3_-N, NO_2_-N, and NH_4_-N), potassium, calcium, magnesium, and sodium content of the wet deposition on January 24–25 was conducted using ion chromatography (Dionex Integrion RFIC, Thermo Fisher Scientific K.K.).

**Table 2:** Chemical analysis of wet deposition at NBG.

Element	Amount (mg/L)	
	November 1, 2022	January 24–25, 2023
Total Carbon	2.7	3.9
Total organic Carbon	2.5	3.3
Inorganic Carbon	0.16	0.60
Inorganic Phosphorus (PO_4_-P)	< 0.008	0.018
Inorganic Nitrogen (NO_3_-N, NO_2_-N, NH_4_-N)	0.12	0.192
Potassium (K)	0.089	0.53
Calcium (Ca)	0.66	0.78
Magnesium (Mg)	0.14	0.70
Sodium (Na)	0.47	6.6
Chloride ion (Cl^−^)	0.085	12
Sulfate ion (SO_4_^2−^)	0.25	2.4

The values obtained for the wet deposition collected on November 1, 2022 and January 24–25, 2023 are compiled in Table 2, and considered to be the reference values for the wet deposition used in the experiment. It should be noted that nutrient levels in wet deposition vary seasonally^20–22^. Unfortunately, we did not analyze the chemical components of wet deposition used in experiment #3 and did not quantify seasonal or annual changes in nutrient status.

### Plant analysis

Whole-plant samples were analyzed for dry weight, total phosphorus, and total nitrogen.

We used the dry ashing method^23,24^ to prepare plant material for analysis. Plant samples in crucibles were heated at 550 °C for 1 h. The samples were then dissolved in 2 M H_2_SO_4_, and the solution was shaken for at least 16 h. The solution was filtered, and the filtrate was diluted tenfold by adding Tris–HCl buffer (pH 8.0). Phosphorus content was determined using the molybdenum blue method^25^.

We performed nitrogen analysis of plant material with a MICRO CORDER JM 10 elemental analyzer (J-Science Lab Co., Ltd., Kyoto, Japan) at the Center for Instrumental Analysis of Gunma University.

### Growth experiments

The *F. japonica* seedlings used in the growth experiments were grown from seeds collected in Gotemba City, Shizuoka in 2017. The collection site is managed by the Numazu Civil Engineering Office, and we received confirmation from the office that no permission was required to collect *F. japonica* seeds. *F. japonica* was identified by Masaki Tateno. A voucher was deposited in the herbarium of the University Museum, the University of Tokyo (No. TI00256544). After collection, *F. japonica* seeds were stored in a refrigerator at NBG until the start of the experiment. For germination the seeds were soaked in a small amount of distilled water and then germinated in an incubator (FLI-2000 or FLI-2010, EYELA, Tokyo, Japan).

The number of samples at the start of the experiment is given as “(*n* =)” at the end of the title of the following experiment, but several of them died during the cultivation process. The number of samples at the end of the experiment is represented by the *n* value in figure legend and the raw data of dry weight in Supplementary Material.

#### Experiment #1: Plant growth and phosphorus absorption in volcanic soils with acidic deposition (*n *= 30)

Two types of volcanic soils were prepared, FJ, a young volcanic soil, and KN, an older, weathered volcanic soil (Table 1). Two flowerpots (0.024 m^2^ each) were prepared for each type; one was treated with 1.0 × 10^–5^ M H_2_SO_4_ (pH 4.7–4.8) and the other with distilled water (DW). The pH of the sulfuric acid solution was confirmed to be within the range corresponding to that of wet deposition collected at NBG (pH 4.6–5.1), used as reference in these experiments. Each flowerpot received 1050 mL sulfuric acid solution or DW per week, as in experiment #3 (see experiment #3). Thirty *F. japonica* seedlings per flowerpot were cultivated in an incubator (FLI-2000 or FLI-2010, EYELA) at 25 °C with a 14 h photoperiod for 92 days. After cultivation, the plants were dried at 80 °C for at least 2 days before dry weight and phosphorus content were measured.

#### Experiment #2: Differences in plant growth on volcanic soil with and without total (wet + dry) deposition (*n* = 50)

KN volcanic soil was placed in two 0.089 m^2^ flowerpots in an open space at NBG. A roof covering of transparent PVC film was set up, and *F. japonica* was grown in covered and uncovered conditions (Supplementary Fig. S2). Fifty *F. japonica* seedlings per flowerpot were cultivated for 88 days (09-May-2022 to 04-Aug-2022). The experiment was initially started with 50 *F. japonica* seedlings per flowerpot, but over 15 died because they could not adapt to the outdoor environment. Total wet deposition at NBG during the growing experiment was 787.5 mm. DW was added when the soil became dry during the cultivation. After cultivation, the plants were dried at 80 °C for at least 2 days before dry weight; phosphorus content, and nitrogen content were then measured.

#### Experiment #3: Effect of wet deposition on the primary succession of plants growing in volcanic soils (*n* = 30)

Six types of volcanic soil (SJ, FJ, HR, NS, KN and KR) were placed in each 0.024 m^2^ flowerpots. Two flowerpots were prepared for each soil type; one received collected wet deposition (WD) and the other received DW. To determine the amount of water needed for the experiment, the annual precipitation in the NBG was measured from November 2018 to October 2019 and determined to be 2261 mm. Based on data, the daily wet deposition was calculated to be 150 mL per flowerpot, per day using Eq. (1):

1 $$\frac{0.024 {\text{ m}}^{2}\times 2261\times {10}^{-3}\text{ m}}{365 {\text{day}}} \cong 149\times {10}^{-6} \frac{{\text{m}}^{3}}{\text{ day}} \cong 150 \frac{\text{mL}}{{\text{day}}} =1050 \frac{\text{mL}}{{\text{we}}{\text{ek}}}$$

Thirty *F. japonica* seedlings per flowerpot were cultivated in an incubator (FLI-2000 or FLI-2010, EYELA) at 25 °C with a 14 h photoperiod for 99 days (31-Aug-2020 to 07-Dec-2020).

We performed several calculations, which are presented below, and the detailed calculation procedure is described in Supplementary Fig. S7. Phosphorus contribution of soil and wet deposition was calculated for each soil type. In addition, we calculated the relative displacement of the dry weight of the *F. japonica* grown under distilled water conditions as 100 and the relative displacement of the phosphorus content of the *F. japonica* when the value of the *F. japonica* seeds as 100. Finally, the relative displacement values of all soil types were pooled to calculate the relative phosphorus contributions of soil and wet deposition in average.

#### Experiment #4: Plant growth with phosphorus and nitrogen fertilization (*n* = 10)

Six types of volcanic soil (SJ, FJ, HR, NS, KN and KR) were used in this experiment. Four treatment groups, DW, nitrogen (N: 10 mM NH_4_NO_3_), phosphorus (P: 10 mM NaH_2_PO_4_), and both nitrogen and phosphorus (NP: N + P) additions were prepared. Five *F. japonica* seedlings were planted per 0.0064 m^2^ flowerpot. Ten *F. japonica* seedlings (two flowerpots) for each nutrient condition (DW, N, P, or NP) were cultivated in an incubator (Incubator; FLI-2000 and FLI-2010, EYELA) at 22 °C with a 10 h photoperiod for 43 days. Once a week, 50 mL of DW, N, P, or NP was added to the soil per flowerpot, and DW was added to all groups when the soil was dry. After cultivation, the plants were dried at 80 °C for at least 2 days, and dry weight was measured. For the DW and N addition conditions, the phosphorus content of the plants was analyzed.

### Statistics

All statistical analyses were performed with EZR^26^ (Saitama Medical Center, Jichi Medical University, Saitama, Japan), a graphical user interface for R (The R Foundation for Statistical Computing, Vienna, Austria). Box-and-whisker plots were produced for data where *n* > 20, and bar charts for *n* < 20. Results are provided as mean ± SD in the bar charts. Intergroup differences were evaluated using a non-parametric Kruskal–Wallis test with a post-hoc Steel–Dwass test, or by two-tailed Mann–Whitney U-test. A *p*-value < 0.05 was considered significant.

### Ethical approval and consent to participate

All experimental research on plants, including the collection of plant and soil material, comply with relevant institutional, national, and international guidelines and legislation.

## Results

### Experiment #1: Plant growth and phosphorus absorption in volcanic soils with acidic deposition

**Figure 2:**
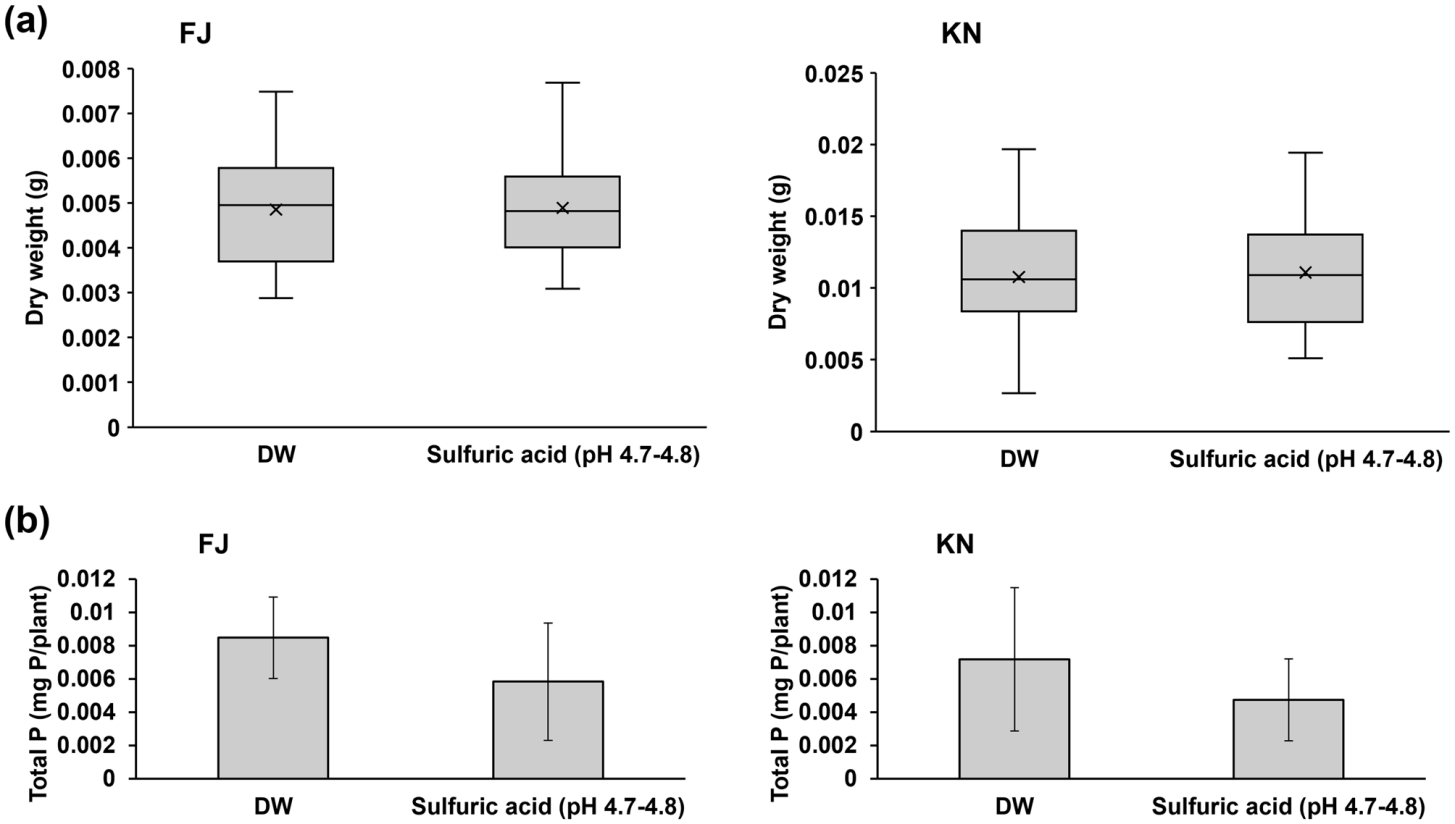
Growth and phosphorus absorption of *Fallopia japonica* on the volcanic soils with application of acid solution of equivalent pH to acidic depositions. (**a**) Dry weight of *F. japonica* (*n* = 25–27). (**b**) Total phosphorus in *F. japonica* (*n* = 8). Bar graphs show mean ± SD. Mann–Whitney U-tests were used. The raw data and *n* are available in Supplementary Table S2.

Acidic solutions with the same pH as wet deposition did not affect the growth or phosphorus content of plants grown in volcanic soils (Fig. 2, Supplementary Fig. S3). No significant differences was found in *F. japonica* growth or phosphorus content among the DW and sulfuric acid solution treatment for both FJ and KN (Fig. 2a,b).

### Experiment #2: Differences in plant growth on volcanic soil with and without total (wet + dry) deposition

**Figure 3:**
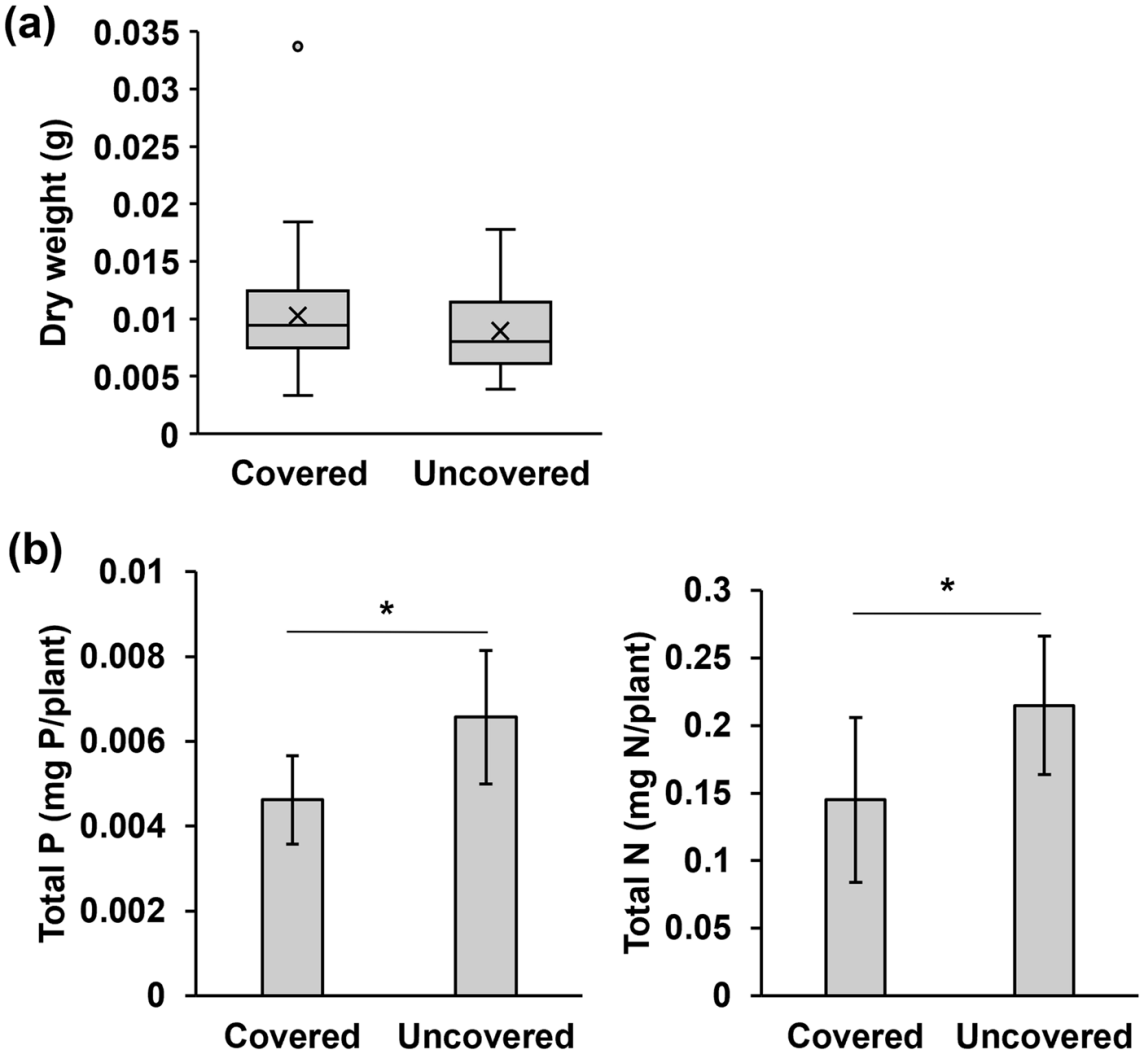
Differences in *F. japonica* growth in covered and uncovered area. (**a**) Dry weight of *F. japonica* (*n* = 32–34). (**b**) Total phosphorus and nitrogen in *F. japonica* (*n* = 8). Bar graphs show mean ± SD. *p* values are *p < 0.05, Mann–Whitney U-test. The raw data and *n* are available in Supplementary Table S3.

When *F. japonica* was grown outdoors at the NBG (Fig. 3, Supplementary Figs. S2, S4), the amount of growth was comparable between plants grown covered and uncovered area (Fig. 3a). However, the phosphorus and nitrogen contents of uncovered plants were significantly higher (approximately 40–50% higher) than those of the covered plants (P: *p* = 0.0208; N: *p* = 0.0207; Fig. 3b).

### Experiment #3: Effect of wet deposition on the primary succession of plants growing in volcanic soils

**Figure 4:**
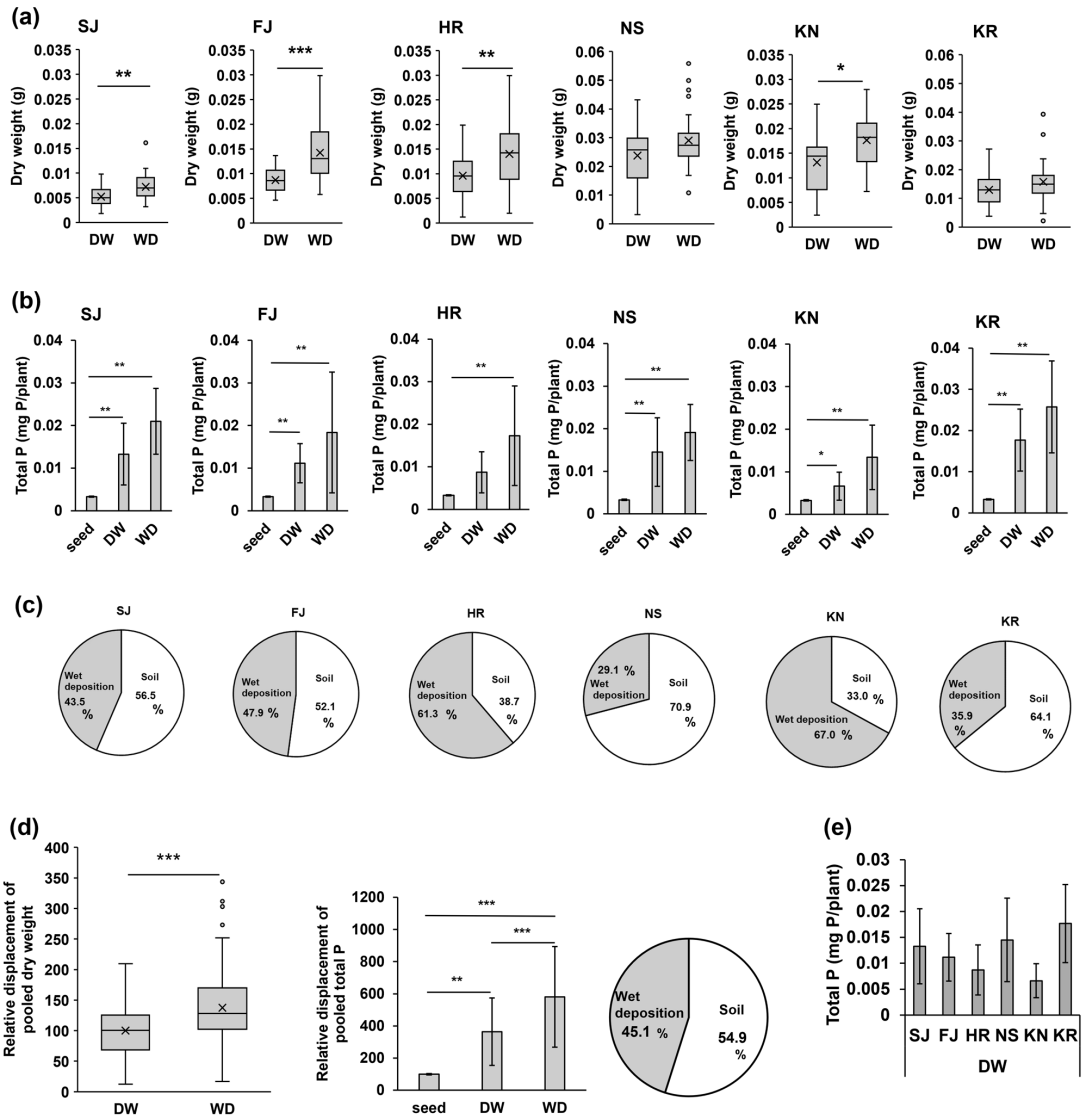
Effect of wet deposition (WD) on the growth of *F. japonica*. (**a**) Dry weight of *F. japonica* (*n* = 25–30). (**b**) Total phosphorus in *F. japonica* (*n* = 8, seed; *n* = 5). (**c**) Contribution of soil and wet deposition to total phosphorus. (**d**) Relative displacement of pooled dry weight (*n* = 168–177), total phosphorus (*n* = 48, seed; *n* = 5), and contribution of soil and wet deposition to phosphorus. (**e**) Total phosphorus in *F. japonica* grown with the addition of DW. Bar graphs show mean ± SD. Mann–Whitney U-test were used for (**a,d**). Kruskal–Wallis with post-hoc Steel–Dwass tests were used for (**b,d,e**). *p* values are *p < 0.05, **p < 0.01, ***p < 0.001. The raw data and *n* are available in Supplementary Table S4.

In experiment 3, the effects of wet deposition using six volcanic soils of different ages were investigated to determine if wet deposition can function as a source of phosphorus. In all soils, the addition of wet deposition (WD), rather than DW, tended to improve *F. japonica* growth and increase the phosphorus content in the plant (Fig. 4a,b, Supplementary Fig. S5). Calculating the relative displacements and pooling the results for all soils revealed that wet deposition increased the growth (Fig. 4d, DW vs. WD, *p* = 2.26 × 10^–10^) and phosphorus content (Fig. 4d, DW vs. WD, *p* = 7.80 × 10^–4^; DW vs. seed, *p* = 0.00169; WD vs. seed, *p* = 7.62 × 10^–4^) of *F. japonica*.

Next, we estimated the relative contributions of soil and wet deposition to the phosphorus contents of the plants. If the average phosphorus content of seeds is 100, the average relative phosphorus content of *F. japonica* grown with the addition of DW is 364 and that of WD is 581 (Fig. 4d). The phosphorus obtained by the *F. japonica* could have two possible sources: in soil and in wet deposition. In other words, the phosphorus obtained from soil and wet deposition was estimated to be 264 (= 364–100) and 217 (= 481–264), respectively. The total phosphorus obtained from soil and wet deposition is estimated to be 481 (= 264 + 217 or = 581–100). From the above, the phosphorus from soil was calculated to be 54.9% (= 264 × 100/481) and from wet deposition 45.1% (= 217 × 100/481) (Fig. 4d). Although the contributions varied with soil type (Fig. 4c), about half of the phosphorus absorbed by the plants was attributed to the soil, and the other half was from wet deposition (Fig. 4d).

No significant difference was found between the phosphorus contents of *F. japonica* grown in the six soil types in the case of DW addition (Fig. 4e). In other words, there was no tendency for younger soils to absorb more phosphorus from the soil by *F. japonica* (Table 1, Fig. 4e).

### Experiment #4: Plant growth with phosphorus and nitrogen fertilization

**Figure 5:**
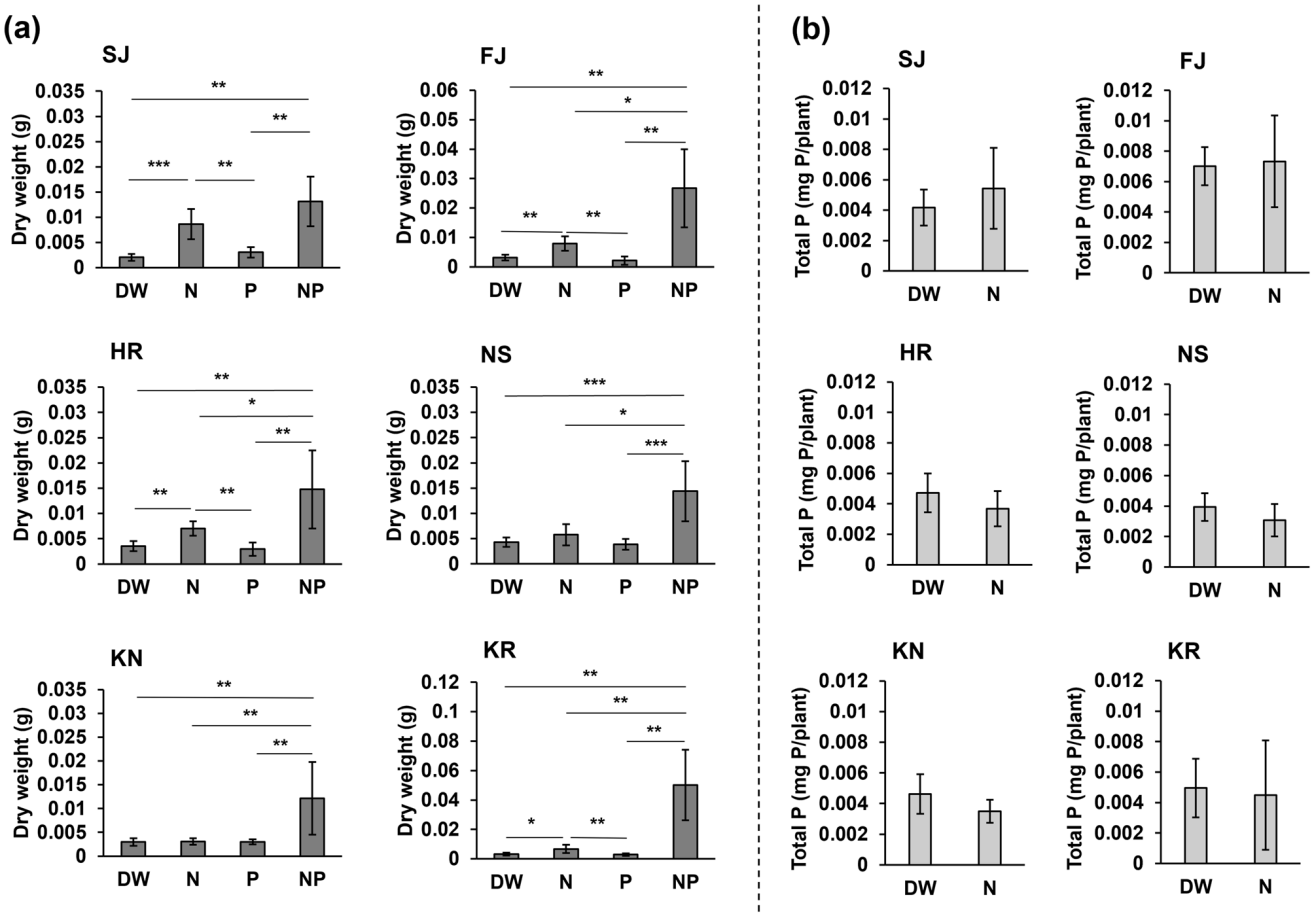
*F. japonica* growth with added phosphorus and nitrogen. (**a**) Dry weight of *F. japonica* (*n* = 7–10). (**b**) Total phosphorus in *F. japonica* (*n* = 8). Bar graphs show mean ± SD. Kruskal–Wallis with post-hoc Steel–Dwass tests were used for (**a**). Mann–Whitney U-tests were used for (**b**). *p* values are *p < 0.05, **p < 0.01, ***p < 0.001. The raw data and *n* are available in Supplementary Table S5.

In SJ, FJ, HR, and KR soils, dry weight more than doubled growth improvement was found with the addition of nitrogen (DW vs. N; SJ: *p* = 9.03 × 10^–4^; FJ: *p* = 0.00815; HR: *p* = 0.00252; KR: *p* = 0.0133; Fig. 5a, Supplementary Fig. S6). In all volcanic soils, a particularly significant (at least threefold) growth improvement was found with the addition of both nitrogen and phosphorus (DW vs. NP; SJ: *p* = 0.00215; FJ: *p* = 0.00186; HR: *p* = 0.00558; NS: *p* = 9.03 × 10^–4^; KN: *p* = 0.00299; KR: *p* = 0.00136; Fig. 5a, Supplementary Fig. S6). In some of the soils (SJ, FJ, HR, and KR), plant growth was improved by nitrogen, so we also tested whether nitrogen addition promoted phosphorus absorption. The results showed no significant difference in the phosphorus content of plants treated with distilled water or excess nitrogen (Fig. 5b). These results suggest that the addition of nitrogen to immature soil did not promote phosphorus absorption.

## Discussion

We aimed to investigate whether the phosphorus supplied to *F. japonica* is supplied directly by deposition or dissolved from volcanic soils due to acidic deposition and found the latter to be false.

The acid solution treatment, which simulated wet deposition did not increase the phosphorus uptake of *F. japonica* in young or old soils (Fig. 2). Therefore, the weak acidity of deposition did not cause significant dissolution of absorbed phosphorus in volcanic soils, indicating that the two major sources of phosphorus are from wet deposition and soluble phosphorus in the soil. We also showed that the contributions of wet deposition and soil to phosphorus in *F. japonica* were approximately 50% each (Fig. 4). Therefore, it can be concluded that for the growth of pioneer plants during the early stage of primary succession, the phosphorus supply contribution of wet deposition is equal to that of soil available phosphorus.

Although total (wet + dry) deposition provided phosphorus to the volcanic soil (Fig. 3), this study was unable to verify what proportions of dry and wet deposition provides more plant-available phosphorus individually. It has been reported that dry deposition supplies more phosphorus than wet deposition in the Mediterranean coast of Israel^27^. However, precipitation in Japan is much higher than that in Mediterranean countries^28^. Therefore, it cannot be concluded that dry deposition is more important in Japan. The effect of dry deposition in Japan’s humic climate is a topic for future work.

The addition of both nitrogen and phosphorus significantly improved the growth of *F. japonica* in the six soils used in this study (Fig. 5). From this, it was clear that the six volcanic soils were deficient in both nitrogen and phosphorus. However, this study did not examine whether nitrogen or phosphorus was relatively more important, and that is a subject for future study. Although wet deposition generally contains phosphorus and nitrogen (Table 2)^8,20–22^, the rapid growth of *F. japonica* in immature volcanic soils requires large amounts of phosphorus and nitrogen (Fig. 5), and it is impossible to rapidly increase the growth rate of *F. japonica* with the nutrient content of wet deposition (Figs. 3, 4). These results suggest that the pioneer species of the early stages of primary succession, *F. japonica,* grows gradually, absorbing a very small amount of phosphorus and nitrogen available through deposition and in volcanic soils.

Typically younger volcanic soils have more soluble phosphorus than older soils^6^, suggesting that plants can more easily utilize phosphorus in younger volcanic soils. However, the values of active Al, active Fe, which adsorb phosphorus strongly, or the phosphate absorption coefficient did not increase with soil age (Fig. 1, Table 1). Most soils had similar properties. These results indicate that in our case volcanic soils of various ages have similar phosphorus adsorption properties. In fact, no difference was found in the phosphorus content of *F. japonica* across all soils (Fig. 4). Nitrogen addition improved growth slightly in soils with low values for active Al, active Fe, and phosphate absorption coefficients, such as SJ, FJ, HR, and KR, but it did not increase the phosphorus absorption by the plants (Fig. 5). One possible explanation of these results is that *F. japonica* likely absorbs the majority of available phosphorus, even under nitrogen deficiency. Note that this study did not accurately test the effect of soil age because the soils differed in types (pumice, tuff, etc., Table 1). At least, it is suggested that young volcanic soil does not always promote plant growth and that old volcanic soil does not always thoroughly suppress plant growth, which could be interesting to investigate in the future.

It is important to note that wet deposition contains a variety of elements other than nitrogen and phosphorus (Table 2). Potassium, calcium, and magnesium are essential elements and may be a significant source of plants, and wet deposition may be a source of various elements in oligotrophic volcanic soils during the early stages of primary succession.

In this study, we showed that, in the early stage of the primary succession in volcanic soils, *F. japonica* grows gradually, using phosphorus supplied by deposition and soil. However, how phosphorus came to be included in deposition still needs to be determined. Most phosphorus in the ecosystem exists in soil and sea water, with very little present in the atmosphere^29^. Because wet deposition also contains organic carbon (Table 2), it is possible that plant materials (pollen and fragmented litter from nearby forests) are being blown up by the wind. It has been reported that the atmosphere contains plant material, which provides a little phosphorus^30^. Plant material may therefore be the important source of atmospheric phosphorus. The determination of plant-derived constituents in deposition is currently under verification and is a subject for future work.

### Supplementary Information

Supplementary Information.

## Data Availability

All data are included in this published article and its Supplementary Information files.
