# Supplementary material for: Deposition is a phosphorus source for Fallopia japonica during early-stage primary succession
Source: Sci Rep. 2023 Sep 25;13:16028. doi: 10.1038/s41598-023-42935-z (PMC10520011; doi:10.1038/s41598-023-42935-z)
Supplement: Supplementary file 1 — Supplementary Information. [file 41598_2023_42935_MOESM1_ESM.pdf]

Supplementary Material

Deposition is a phosphorus source for *Fallopia japonica* during early-stage primary succession

Sae Katayama[1]*, Koichiro Sawakami[1], and Masaki Tateno[1]

[1]Nikko Botanical Garden, Graduate School of Science, University of Tokyo, Japan

* Corresponding author

E-mail: saekatayama.nbg@gmail.com

Tel: +81-288-54-0206

Fax: +81-288-54-3178

Supplementary Figure

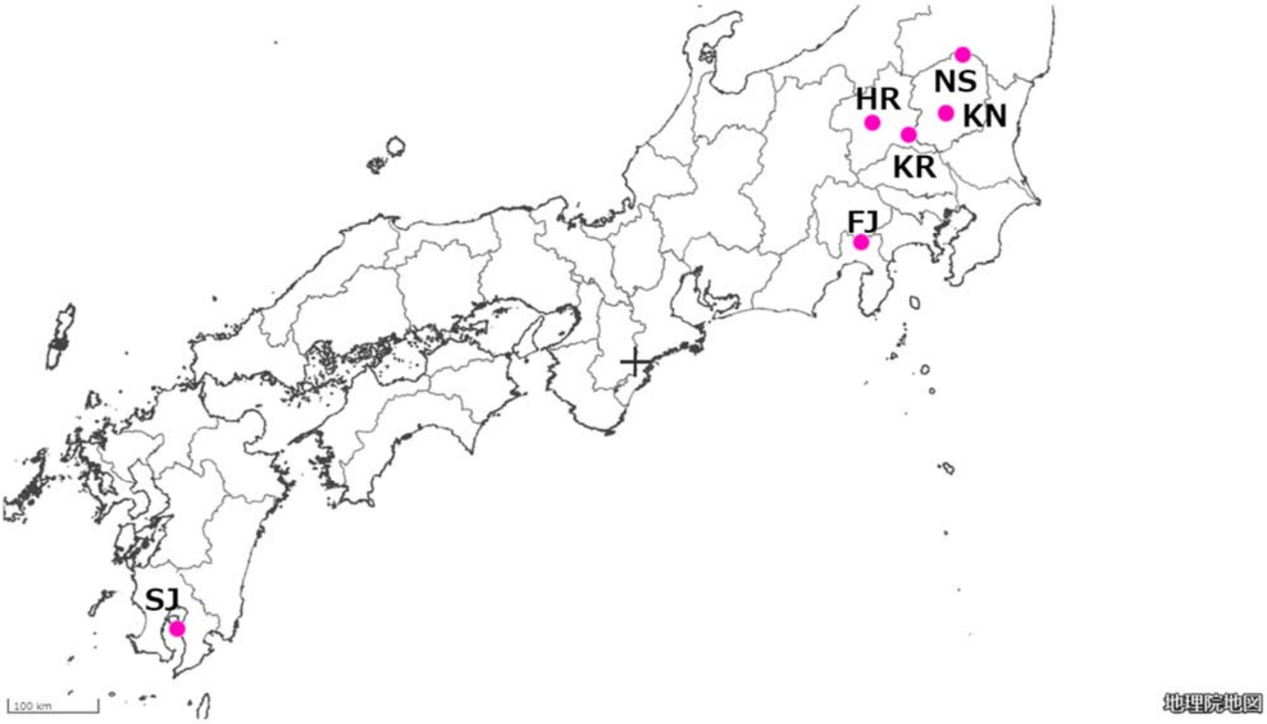

Fig. S1 Approximate distribution of soils used in this study. Sakurajima (SJ), Fuji (FJ), Haruna (HR), Nasu (NS), Kanuma (KN) and Kiryu (KR) soils were used in this study. This figure was created by processing a map from the Geospatial Information Authority of Japan (https://maps.gsi.go.jp/).

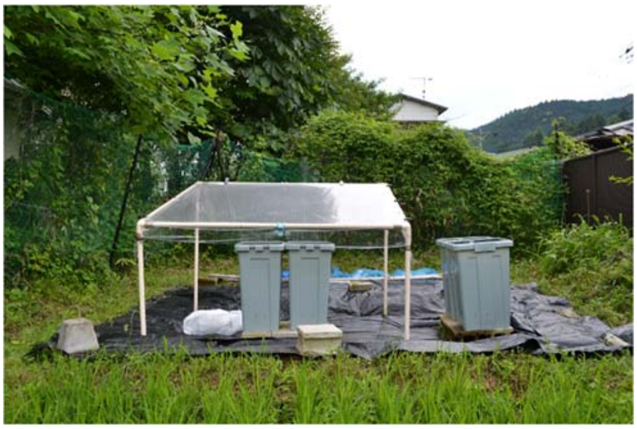

Fig. S2 Photograph of field conditions for experiment #2.

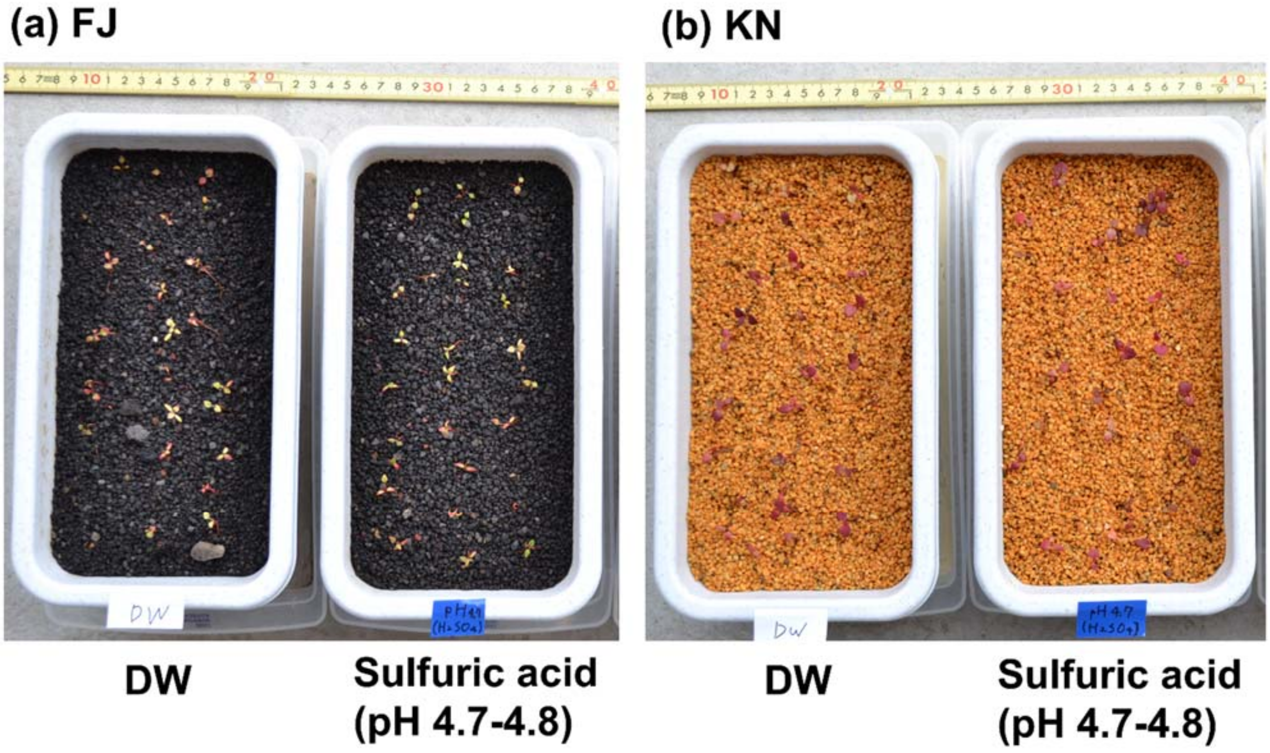

Fig. S3 Photograph of *F. japonica* seedlings at the end of experiment #1. (a) Fuji soil (FJ), (b) Kanuma soil (KN).

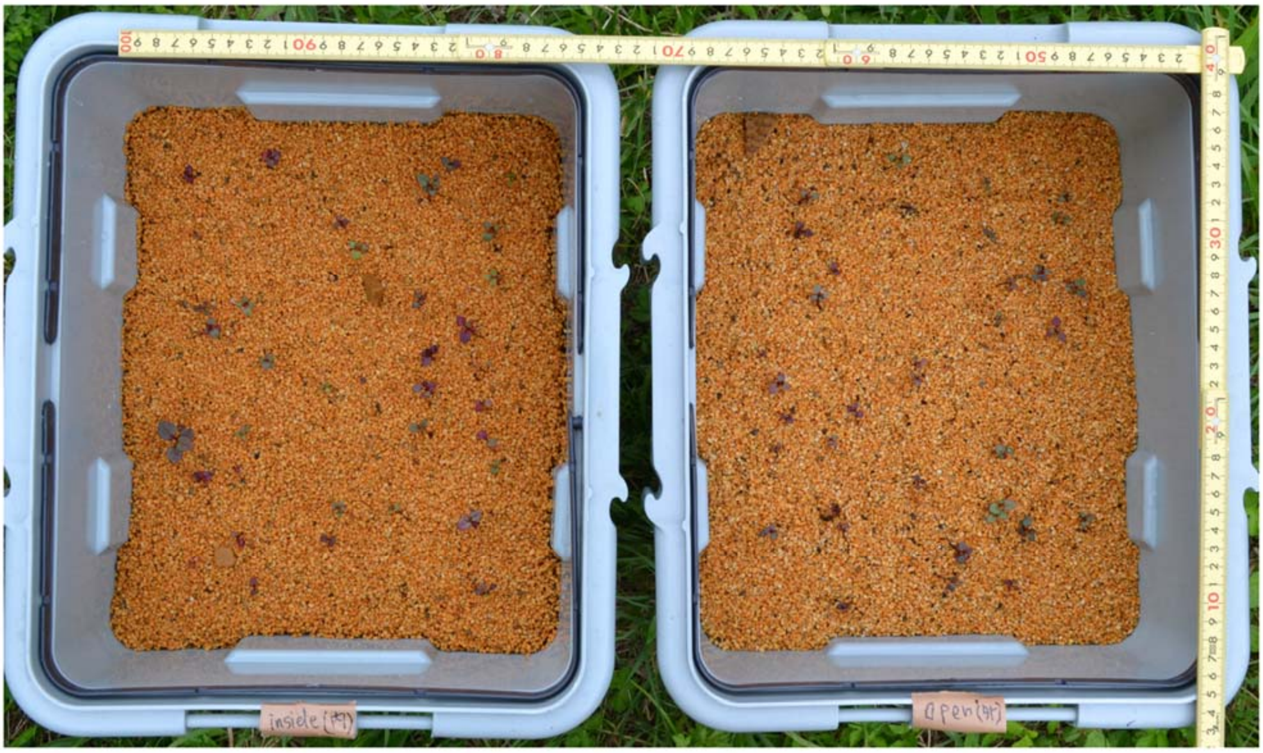

Covered Uncovered

Fig. S4 Photograph of *F. japonica* seedlings at the end of experiment #2.

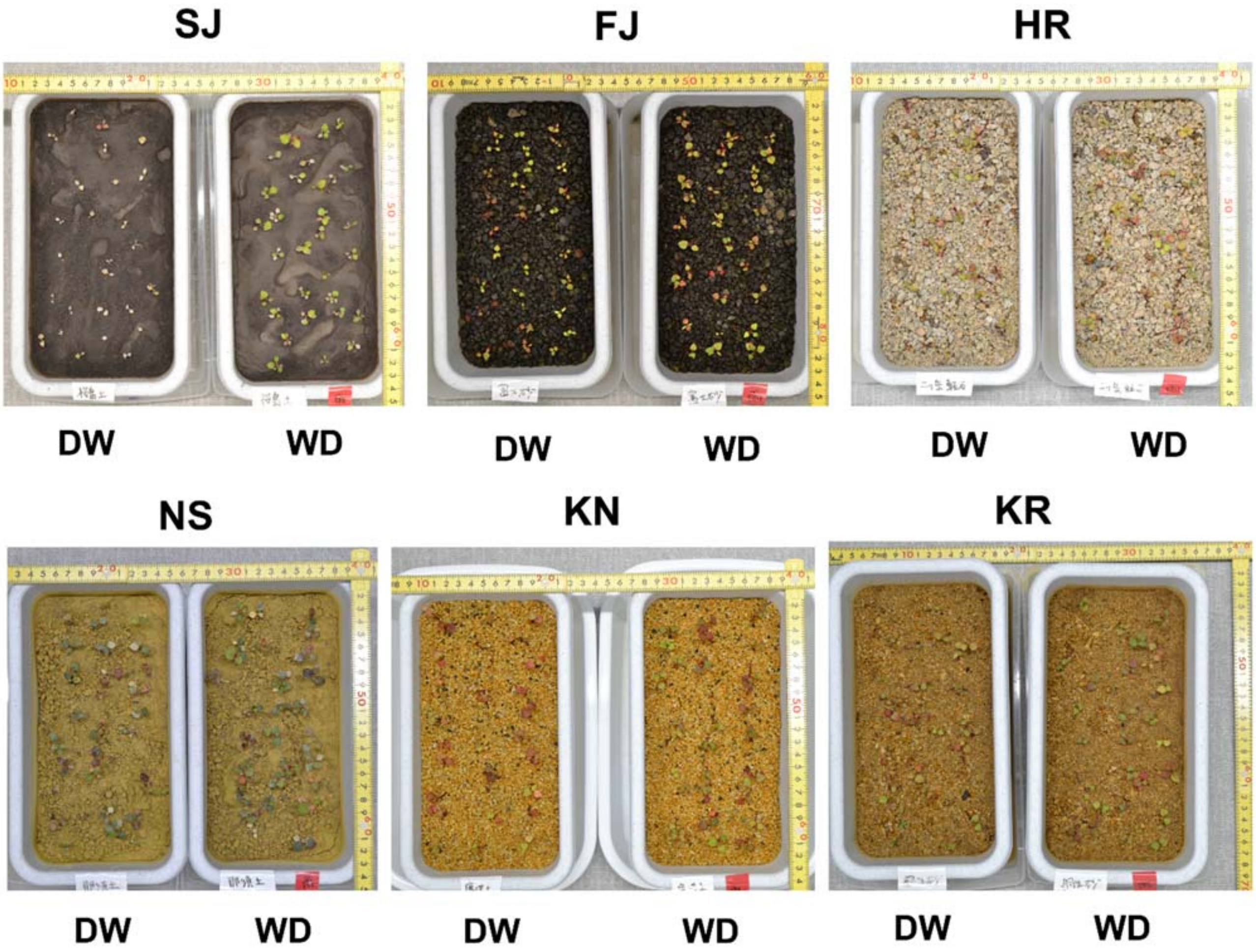

Fig. S5 Photograph of *F. japonica* seedlings at the end of experiment #3.

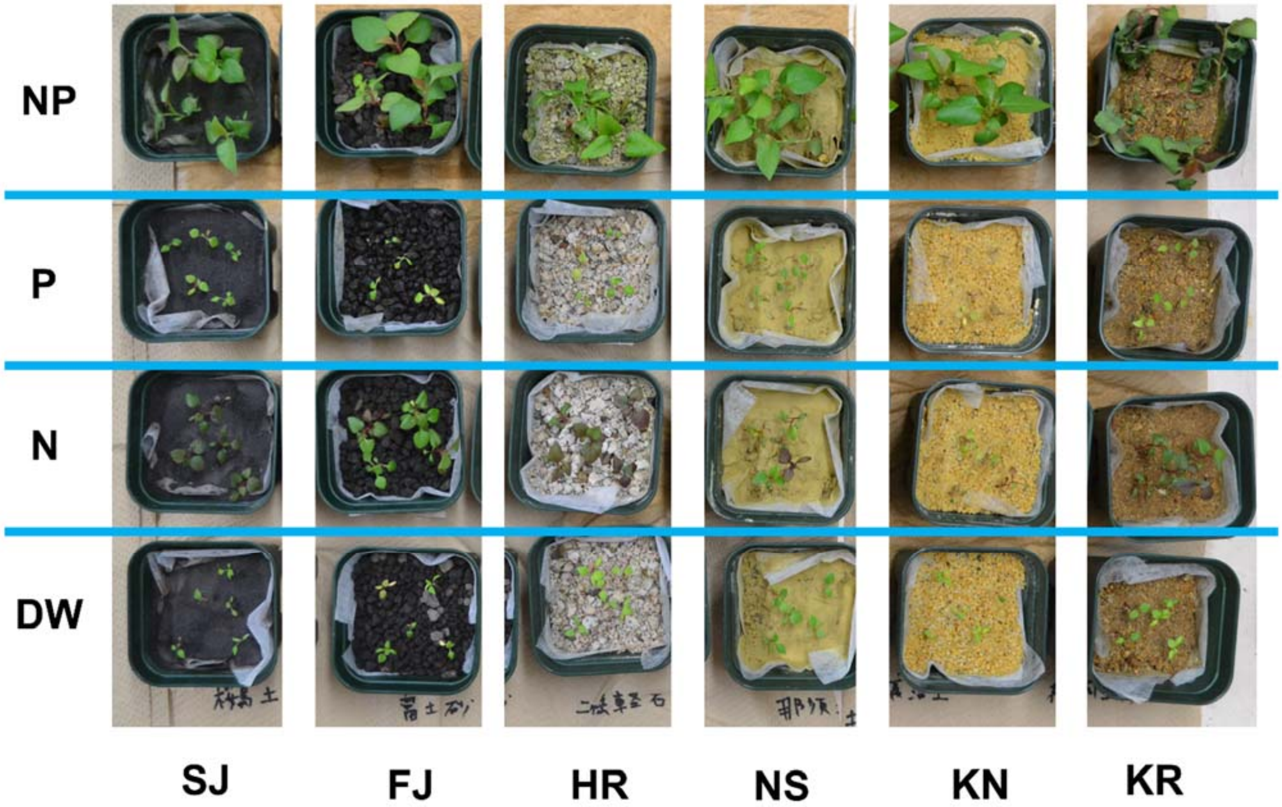

Fig. S6 Photograph of *F. japonica* seedlings at the end of experiment #4.

(a) How to calculate the data in Fig. 4c

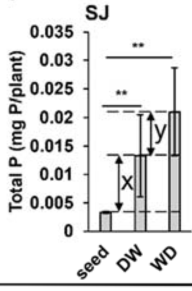

$$\text{P supply contribution of soil} = \frac{100x}{x+y}$$

$$\text{P supply contribution of wet deposition} = \frac{100y}{x+y}$$

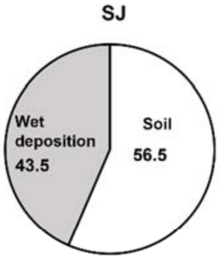

(b) How to calculate the data in Fig. 4d

Dry weight of plant (SJ)		
	DW	WD
1	d1	r1
2	d2	r2
:	:	:
Average	d	r

Relative displacement of Total P (SJ)		
	DW	WD
1	$\frac{100d1}{d}$	$\frac{100r1}{d}$
2	$\frac{100d2}{d}$	$\frac{100r2}{d}$
:	:	:
Average	100	r′

Pooling 6 soils data

SJ, FJ, HR, NS, KN, KR

Relative displacement of pooled Total P			
	DW	WD	Soil type
1	$\frac{100d1}{d}$	$\frac{100r1}{d}$	SJ
2	$\frac{100d2}{d}$	$\frac{100r2}{d}$	SJ
:	:	:	:
:	:	:	FJ
:	:	:	:
Average	100	r″	

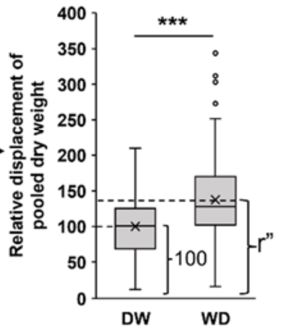

Total P of plant (SJ)			
	seed	DW	WD
1	S1	D1	R1
2	S2	D2	R2
:	:	:	:
Average	S	D	R

Relative displacement of Total P (SJ)			
	seed	DW	WD
1	$\frac{100S1}{S}$	$\frac{100D1}{S}$	$\frac{100R1}{S}$
2	$\frac{100S2}{S}$	$\frac{100D2}{S}$	$\frac{100R2}{S}$
:	:	:	:
Average	100	D′	R′

Pooling 6 soils data

SJ, FJ, HR, NS, KN, KR

Relative displacement of pooled Total P				
	seed	DW	WD	Soil type
1	$\frac{100S1}{S}$	$\frac{100D1}{S}$	$\frac{100R1}{S}$	SJ
2	$\frac{100S2}{S}$	$\frac{100D2}{S}$	$\frac{100R2}{S}$	SJ
:	:	:	:	:
:		:	:	FJ
:		:	:	:
Average	100	D″	R″	

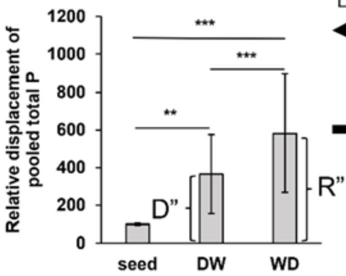

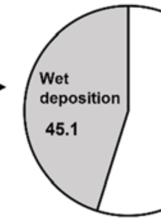

$$\text{contribution of soil} = \frac{100(D''-100)}{(R''-100)}$$

$$\text{contribution of wet deposition} = \frac{100(R''-D'')}{(R''-100)}$$

Fig S7 How to calculate the data in (a) Fig. 4c, and (b) Fig. 4d. Experiment #1 (Fig. 2) showed that the pH of wet deposition does not excessively dissolve adsorbed phosphorus in volcanic soils. Therefore, phosphorus supply from wet deposition and that from soil were calculated assuming that the phosphorus gain by wet deposition-treatment minus that gain by DW-treatment was the

contribution of wet deposition, and the phosphorus gain by DW-treatment minus that contained in seed was the contribution of soil. In experiment #3, (a) Fig 4c, the difference "*x*" between the average phosphorus content in *F. japonica* seeds and that in plants grown with the addition of distilled water was considered soil-derived phosphorus. The difference "*y*" between the average phosphorus content in plants grown with the addition of wet deposition and those grown with distilled water was considered as the phosphorus derived from wet deposition. Because the only sources of phosphorus in experiment #3 were wet deposition and soil, the relative phosphorus contributions of wet deposition and soil were calculated from the values of *x* and *y*. In (b) Fig. 4d, the relative displacement was calculated by taking the average dry weight value of the *F. japonica* under the condition of distilled water addition as 100. Then, the relative displacement was derived by taking the average total P in plant value of the *F. japonica* seed as 100. Finally, the relative displacement values of all soil types were pooled to calculate the relative phosphorus contributions of soil and wet deposition in average.

Supplementary Table

Table S1. Raw data of Fig. 1

	Active Al (g/kg)	Active Fe (g/kg)	Phosphate absorption coefficient (g/kg)	Total P (g/kg)	Total N (g/kg)	Total C (g/kg)
SJ	1	5.9	4.2	0.6	0.2	0.4
FJ	2.2	7.6	6.2	1.23	0.2	0.9
HR	2.6	8.9	7.4	0.38	0.09	0.5
NS	4.2	28.6	11.5	0.51	0.1	0.7
KN	121	11.9	26.9	0.53	0.6	2.9
KR	1.6	5.6	10	0.23	0.1	0.5

Table S2. Raw data of Fig. 2

(a) Dry weight of plant

	Dry weight（g）			
	FJ		KN	
	DW	Sulfuric acid (pH 4.7-4.8)	DW	Sulfuric acid (pH 4.7-4.8)
1	0.00495	0.00482	0.01531	0.00996
2	0.00711	0.00507	0.00867	0.01373
3	0.00476	0.00505	0.01589	0.01944
4	0.00564	0.00746	0.01801	0.0154
5	0.00749	0.00516	0.0111	0.01109
6	0.0051	0.00501	0.01302	0.00789
7	0.00481	0.00769	0.01061	0.00648
8	0.00496	0.00354	0.01195	0.00679
9	0.00716	0.00513	0.01016	0.016
10	0.00415	0.00442	0.0124	0.00638
11	0.00498	0.00369	0.00876	0.0056
12	0.00321	0.0059	0.01401	0.01372
13	0.00604	0.00627	0.01452	0.0179
14	0.00289	0.00309	0.01967	0.01089
15	0.00548	0.00479	0.01288	0.01365
16	0.00576	0.00462	0.00838	0.01447
17	0.00619	0.00551	0.00987	0.01252
18	0.00323	0.00607	0.01444	0.01082
19	0.00584	0.00325	0.00915	0.00948
20	0.00288	0.00483	0.01136	0.01232
21	0.00304	0.00403	0.00884	0.01353
22	0.00508	0.00413	0.00765	0.00511
23	0.00449	0.00586	0.00453	0.00861
24	0.00294	0.00343	0.00594	0.00736
25	0.00385	0.0044	0.00497	0.00799
26	0.00422	0.00397	0.00614	
27			0.00266	
Average	0.00486	0.00489	0.01077	0.01109
sd	0.00135	0.00117	0.00414	0.00394

(b) Total P of plant

	Total P (mg/plant)			
	FJ		KN	
	DW	Sulfuric acid (pH 4.7-4.8)	DW	Sulfuric acid (pH 4.7-4.8)
1	0.00720	0.00205	0.01219	0.00443
2	0.01208	0.00249	0.00393	0.00687
3	0.00698	0.00770	0.00493	0.00953
4	0.00953	0.00804	0.00632	0.00427
5	0.01200	0.00560	0.00643	0.00499
6	0.00765	0.00211	0.01546	0.00272
7	0.00654	0.01203	0.00438	0.00299
8	0.00582	0.00665	0.00382	0.00205
Average	0.00848	0.00583	0.00718	0.00473
sd	0.00245	0.00352	0.00430	0.00246

Table S3. Raw data of Fig. 3

(a) Dry weight of plant

	Dry Weight (g)	
	Covered	Uncovered
1	0.00977	0.01675
2	0.01259	0.00897
3	0.01046	0.01155
4	0.01249	0.01105
5	0.01196	0.01778
6	0.01234	0.01329
7	0.00746	0.01400
8	0.01258	0.00980
9	0.00663	0.00928
10	0.00810	0.01237
11	0.01181	0.01144
12	0.00951	0.00749
13	0.00937	0.00824
14	0.00747	0.00622
15	0.00829	0.01234
16	0.00521	0.00799
17	0.00980	0.01187
18	0.00744	0.01089
19	0.00589	0.00992
20	0.00770	0.00785
21	0.01315	0.00480
22	0.00504	0.00567
23	0.00871	0.00794
24	0.00824	0.00764
25	0.01351	0.00585
26	0.00818	0.00466
27	0.01345	0.00575
28	0.01111	0.00500
29	0.00332	0.00649
30	0.00579	0.00806
31	0.01842	0.00660
32	0.03370	0.00563

33		0.00680
34		0.00388
Average	0.01030	0.00894
sd	0.00531	0.00343

(b) Total P and N of plant

	Total P (mg/plant)	
	Covered	Uncovered
1	0.00493	0.00639
2	0.00595	0.00890
3	0.00529	0.00524
4	0.00286	0.00815
5	0.00326	0.00432
6	0.00511	0.00687
7	0.00476	0.00529
8	0.00480	0.00744
Average	0.00462	0.00657
sd	0.00104	0.00157

	Total N (mg/plant)	
	Covered	Uncovered
1	0.13222	0.25194
2	0.23353	0.22219
3	0.10979	0.28046
4	0.24401	0.23902
5	0.13686	0.25363
6	0.12931	0.16927
7	0.08817	0.13568
8	0.08649	0.16695
Average	0.14505	0.21489
sd	0.06096	0.05136

Table S4. Raw data of Fig. 4

(a) Dry weight of plant

	Dry weight (g)					
	SJ		FJ		HR	
	DW	WD	DW	WD	DW	WD
1	0.00450	0.01613	0.00875	0.01165	0.00149	0.00199
2	0.00756	0.01038	0.01208	0.02028	0.00372	0.00512
3	0.00566	0.01098	0.01082	0.01502	0.00119	0.00875
4	0.00607	0.01023	0.00843	0.01820	0.00879	0.00662
5	0.00678	0.00598	0.00912	0.01194	0.00750	0.00341
6	0.00702	0.00746	0.00706	0.01013	0.00455	0.01850
7	0.00184	0.00632	0.00709	0.01452	0.01180	0.01378
8	0.00684	0.00519	0.00892	0.01004	0.01098	0.01794
9	0.00385	0.00921	0.00952	0.00576	0.00571	0.01316
10	0.00979	0.00617	0.00918	0.01303	0.00605	0.01448
11	0.00648	0.00681	0.00556	0.01957	0.01203	0.00902
12	0.00674	0.00427	0.00652	0.01491	0.01902	0.01627
13	0.00467	0.00320	0.01163	0.02985	0.01306	0.02419
14	0.00619	0.00737	0.00720	0.01227	0.00805	0.01518
15	0.00391	0.00518	0.01366	0.01338	0.00987	0.01085
16	0.00499	0.00375	0.00708	0.02372	0.00834	0.00876
17	0.00343	0.00567	0.00747	0.00996	0.01312	0.02412
18	0.00225	0.00742	0.01297	0.01806	0.00951	0.01110
19	0.00479	0.00781	0.00803	0.01199	0.00938	0.01289
20	0.00296	0.00532	0.00524	0.01624	0.00957	0.02287
21	0.00657	0.00461	0.01033	0.00670	0.01267	0.02087
22	0.00574	0.00552	0.01289	0.02043	0.01988	0.02994
23	0.00289	0.00539	0.00623	0.02172	0.01301	0.01549
24	0.00429	0.00744	0.00886	0.00610	0.00530	0.01423
25	0.00483	0.00672	0.00553	0.01030	0.01067	0.01101
26		0.00946	0.01259	0.01209	0.00829	0.01485
27		0.00721	0.00461	0.00687	0.01094	0.01827
28		0.00901	0.00593	0.01874	0.01441	0.01507
29		0.00931		0.00939		0.00802
30		0.00715				
Average	0.00523	0.00722	0.00869	0.01424	0.00960	0.01403

sd	0.00185	0.00261	0.00260	0.00572	0.00444	0.00651

	Dry weight (g)					
	NS		KN		KR	
	DW	WD	DW	WD	DW	WD
1	0.03861	0.02907	0.01603	0.01825	0.02068	0.00681
2	0.04089	0.02774	0.01662	0.01905	0.01383	0.01582
3	0.02775	0.02253	0.01237	0.02796	0.01381	0.02372
4	0.02947	0.01794	0.01579	0.01036	0.01916	0.01724
5	0.01608	0.01081	0.01557	0.01431	0.02151	0.01547
6	0.03395	0.02633	0.01369	0.00723	0.01570	0.03933
7	0.01136	0.04989	0.01609	0.02599	0.01321	0.01574
8	0.02992	0.02266	0.01516	0.01868	0.01085	0.01498
9	0.01601	0.04659	0.02174	0.02754	0.02720	0.01342
10	0.01649	0.02750	0.00774	0.01161	0.00603	0.01485
11	0.02509	0.02713	0.02322	0.02491	0.01124	0.01200
12	0.04314	0.03799	0.01519	0.02206	0.01671	0.01864
13	0.02644	0.03482	0.01077	0.01970	0.01087	0.01130
14	0.03266	0.02812	0.01736	0.01340	0.01298	0.02285
15	0.02750	0.04444	0.01565	0.02247	0.01547	0.01769
16	0.01398	0.05583	0.01181	0.01516	0.00870	0.00811
17	0.01777	0.02436	0.00733	0.02015	0.00946	0.03223
18	0.02855	0.03588	0.01569	0.01926	0.00660	0.02344
19	0.01328	0.02953	0.02115	0.01309	0.01638	0.02236
20	0.01637	0.02650	0.00648	0.01402	0.00373	0.00508
21	0.02668	0.02441	0.00724	0.01904	0.00743	0.01777
22	0.01079	0.01869	0.01821	0.01478	0.00975	0.01207
23	0.02174	0.02487	0.02497	0.01208	0.00522	0.01461
24	0.04157	0.03048	0.00900	0.02664	0.01969	0.01391
25	0.02202	0.01689	0.01118	0.01669	0.00879	0.01114
26	0.00320	0.02017	0.01255	0.01469	0.01792	0.01440
27	0.00687	0.02910	0.00588	0.01327	0.01084	0.01769
28	0.02764	0.02389	0.00454	0.01861	0.00723	0.01341
29		0.02694	0.00244	0.01100	0.01531	0.00476
30		0.02888	0.00312			0.00216
Average	0.02378	0.02900	0.01315	0.01766	0.01298	0.01577
sd	0.01063	0.00991	0.00587	0.00551	0.00555	0.00768

(b) Total P of plant

	Total P (mg/plant)						
		SJ		FJ		HR	
	seed	DW	WD	DW	WD	DW	WD
1	0.00325	0.02025	0.02674	0.02051	0.00679	0.00482	0.00352
2	0.00337	0.01050	0.03362	0.00925	0.05116	0.00980	0.00618
3	0.00325	0.01126	0.02131	0.00980	0.00955	0.00221	0.03398
4	0.00313	0.02302	0.02051	0.01332	0.02000	0.00965	0.03061
5	0.00350	0.02156	0.02307	0.01161	0.02025	0.00668	0.02111
6		0.00555	0.00681	0.01224	0.01322	0.01480	0.00937
7		0.00904	0.01760	0.00580	0.01662	0.01606	0.00933
8		0.00507	0.01816	0.00669	0.00937	0.00572	0.02429
Average	0.00330	0.01328	0.02098	0.01115	0.01837	0.00872	0.01730
sd	0.00014	0.00726	0.00773	0.00459	0.01416	0.00483	0.01170

	Total P (mg/plant)					
	NS		KN		KR	
	DW	WD	DW	WD	DW	WD
1	0.02936	0.02483	0.00955	0.01754	0.03004	0.02994
2	0.02015	0.01578	0.01317	0.00714	0.01036	0.01657
3	0.00837	0.02794	0.00437	0.00563	0.01215	0.04714
4	0.01620	0.02126	0.00442	0.02799	0.01994	0.03609
5	0.01142	0.01836	0.00523	0.00809	0.00658	0.02183
6	0.01825	0.02194	0.00701	0.01853	0.01946	0.02108
7	0.00649	0.01638	0.00596	0.00941	0.02177	0.01561
8	0.00588	0.00641	0.00332	0.01293	0.02112	0.01768
Average	0.01451	0.01911	0.00663	0.01341	0.01768	0.02574
sd	0.00806	0.00659	0.00327	0.00756	0.00753	0.01116

(c) Relative displacement of dry weight data

	SJ		FJ		HR	
	DW	WD	DW	WD	DW	WD
1	86.19	308.59	100.69	134.06	15.51	20.72
2	144.74	198.71	138.99	233.39	38.78	53.27
3	108.27	210.04	124.53	172.83	12.39	91.15
4	116.12	195.76	97.05	209.43	91.53	68.93
5	129.67	114.39	104.92	137.45	78.12	35.49
6	134.37	142.75	81.24	116.64	47.42	192.63
7	35.17	120.90	81.58	167.07	122.83	143.49
8	130.81	99.35	102.64	115.58	114.35	186.82
9	73.64	176.28	109.53	66.27	59.46	137.03
10	187.26	118.07	105.64	150.02	62.98	150.75
11	124.00	130.39	63.97	225.24	125.31	93.90
12	128.90	81.75	75.02	171.61	198.05	169.41
13	89.44	61.16	133.90	343.61	135.97	251.92
14	118.49	141.07	82.89	141.20	83.86	158.04
15	74.82	99.05	157.18	154.02	102.73	112.98
16	95.53	71.68	81.49	273.01	86.86	91.21
17	65.67	108.58	85.93	114.64	136.63	251.13
18	43.13	142.03	149.33	207.91	99.02	115.56
19	91.66	149.41	92.42	137.95	97.63	134.20
20	56.64	101.73	60.33	186.87	99.61	238.12
21	125.69	88.26	118.87	77.14	131.91	217.29
22	109.80	105.55	148.31	235.10	207.02	311.75
23	55.34	103.10	71.71	250.04	135.43	161.31
24	82.13	142.41	102.00	70.25	55.23	148.17
25	92.50	128.56	63.60	118.60	111.14	114.68
26		180.99	144.95	139.15	86.32	154.67
27		137.89	53.06	79.05	113.87	190.22
28		172.38	68.23	215.67	150.05	156.92
29		178.12		108.05		83.53
30		136.90				
Average	100.00	138.19	100.00	163.86	100.00	146.05
sd	35.39	49.97	29.98	65.88	46.23	67.76

	NS		KN		KR	
	DW	WD	DW	WD	DW	WD
1	162.35	122.24	121.85	138.76	159.39	52.47
2	171.96	116.66	126.33	144.87	106.61	121.90
3	116.71	94.75	94.04	212.59	106.46	182.83
4	123.95	75.44	120.04	78.80	147.64	132.85
5	67.63	45.47	118.40	108.83	165.80	119.22
6	142.77	110.72	104.07	55.00	120.96	303.08
7	47.76	209.81	122.31	197.61	101.79	121.33
8	125.81	95.31	115.28	142.06	83.59	115.44
9	67.33	195.91	165.28	209.42	209.63	103.45
10	69.33	115.64	58.86	88.30	46.47	114.47
11	105.49	114.09	176.52	189.41	86.61	92.46
12	181.43	159.77	115.52	167.76	128.75	143.64
13	111.21	146.42	81.92	149.75	83.76	87.08
14	137.33	118.24	131.99	101.85	100.03	176.11
15	115.66	186.88	118.96	170.84	119.24	136.31
16	58.80	234.80	89.78	115.28	67.08	62.53
17	74.73	102.44	55.75	153.19	72.94	248.37
18	120.06	150.87	119.31	146.41	50.83	180.63
19	55.84	124.18	160.82	99.56	126.26	172.30
20	68.84	111.42	49.30	106.57	28.73	39.16
21	112.21	102.64	55.03	144.78	57.29	136.92
22	45.39	78.61	138.44	112.35	75.12	93.05
23	91.44	104.58	189.87	91.86	40.20	112.62
24	174.80	128.19	68.46	202.56	151.74	107.23
25	92.60	71.01	85.00	126.93	67.74	85.84
26	13.44	84.81	95.39	111.68	138.09	110.99
27	28.90	122.38	44.72	100.86	83.52	136.30
28	116.23	100.47	34.49	141.48	55.73	103.35
29		113.29	18.52	83.67	118.00	36.67
30		121.43	23.75			16.62
Average	100.00	121.95	100.00	134.24	100.00	121.51
sd	44.70	41.66	44.63	41.87	42.76	59.20

(d) Relative displacement of total P data

		SJ		FJ		HR	
	seed	DW	WD	DW	WD	DW	WD
1	98.50	614.16	810.75	621.78	205.73	146.30	106.68
2	102.24	318.51	1019.53	280.41	1551.39	297.17	187.45
3	98.50	341.37	646.16	297.17	289.55	67.05	1030.20
4	94.76	697.97	621.78	403.85	606.54	292.60	928.09
5	105.99	653.78	699.50	352.04	614.16	202.69	640.06
6		168.43	206.54	371.28	400.79	448.74	284.00
7		274.16	533.57	175.81	504.06	486.85	282.77
8		153.68	550.78	202.85	284.00	173.35	736.42
Average	100.00	402.76	636.07	338.15	557.03	264.34	524.46
sd	4.26	220.13	234.26	139.25	429.48	146.54	354.83

	NS		KN		KR	
	DW	WD	DW	WD	DW	WD
1	890.16	752.97	289.55	531.86	910.90	907.71
2	610.99	478.58	399.28	216.40	314.27	502.51
3	253.65	847.09	132.58	170.68	368.51	1429.37
4	491.35	644.49	134.11	848.85	604.61	1094.36
5	346.18	556.75	158.49	245.36	199.41	662.04
6	553.24	665.11	212.69	561.84	590.12	639.30
7	196.71	496.68	180.72	285.22	660.20	473.33
8	178.27	194.25	100.81	392.18	640.53	536.03
Average	440.07	579.49	201.03	406.55	536.07	780.58
sd	244.53	199.73	99.07	229.33	228.36	338.29

Table S5. Raw data of Fig. 5

(a) Dry weight of plant

	Dry weight (g)							
	Sakurajima (SJ)				Fuji (FJ)			
	DW	N	P	NP	DW	N	P	NP
1	0.00260	0.01394	0.00204	0.01779	0.00417	0.01059	0.00538	0.03938
2	0.00184	0.01002	0.00490	0.00876	0.00326	0.01012	0.00189	0.04416
3	0.00099	0.00560	0.00241	0.02199	0.00335	0.00709	0.00148	0.01989
4	0.00148	0.01116	0.00367	0.01373	0.00177	0.01076	0.00182	0.03693
5	0.00170	0.00766	0.00145	0.00766	0.00170	0.00618	0.00109	0.03719
6	0.00321	0.01198	0.00347	0.00916	0.00371	0.00819	0.00147	0.03059
7	0.00158	0.00629	0.00263	0.01188	0.00334	0.00370	0.00263	0.02756
8	0.00264	0.00749	0.00333	0.01423	0.00284	0.00674	0.00137	0.01364
9	0.00246	0.00463	0.00270		0.00442			0.01381
10	0.00223	0.00777	0.00415					0.00430
Average	0.00207	0.00865	0.00308	0.01315	0.00317	0.00792	0.00214	0.02675
sd	0.00067	0.00301	0.00103	0.00490	0.00094	0.00248	0.00139	0.01326

	Dry weight (g)							
	Haruna (HR)				Nasu (NS)			
	DW	N	P	NP	DW	N	P	NP
1	0.00330	0.00479	0.00560	0.01567	0.00571	0.00447	0.00222	0.02681
2	0.00320	0.00745	0.00354	0.02473	0.00393	0.00777	0.00404	0.01255
3	0.00387	0.00687	0.00314	0.01070	0.00426	0.00565	0.00320	0.01674
4	0.00342	0.00682	0.00231	0.01250	0.00332	0.00855	0.00312	0.01444
5	0.00200	0.00818	0.00170	0.02731	0.00361	0.00542	0.00605	0.00745
6	0.00211	0.00736	0.00241	0.01232	0.00608	0.00411	0.00356	0.01903
7	0.00424	0.00504	0.00189	0.01073	0.00346	0.00848	0.00382	0.01363
8	0.00331	0.00742		0.00408	0.00483	0.00516	0.00514	0.00797
9	0.00491	0.00943			0.00397	0.00248	0.00398	0.01683
10	0.00501				0.00404		0.00363	0.00842
Average	0.00354	0.00704	0.00294	0.01476	0.00432	0.00579	0.00388	0.01439
sd	0.00102	0.00144	0.00134	0.00770	0.00094	0.00209	0.00107	0.00593

	Dry weight (g)							
	Kanuma (KN)				Kiryu (KR)			
	DW	N	P	NP	DW	N	P	NP
1	0.00310	0.00420	0.00359	0.02728	0.00346	0.01297	0.00285	0.06225
2	0.00293	0.00412	0.00220	0.01904	0.00544	0.00596	0.00415	0.09183
3	0.00467	0.00314	0.00233	0.00739	0.00367	0.00570	0.00358	0.04546
4	0.00336	0.00209	0.00293	0.01497	0.00232	0.00682	0.00142	0.04768
5	0.00312	0.00284	0.00301	0.00814	0.00160	0.00802	0.00292	0.04974
6	0.00193	0.00262	0.00330	0.00666	0.00307	0.00877	0.00303	0.07641
7	0.00228	0.00328	0.00338	0.00657	0.00369	0.00503	0.00278	0.03706
8	0.00244	0.00250		0.00729	0.00267	0.00688	0.00343	0.01629
9	0.00277	0.00279			0.00374	0.00492	0.00251	0.02454
10					0.00279	0.00253	0.00205	
Average	0.00296	0.00306	0.00296	0.01217	0.00325	0.00676	0.00287	0.05014
sd	0.00079	0.00071	0.00053	0.00764	0.00104	0.00279	0.00078	0.02391

(b) Total P of plant

	Total P (mg/plant)					
	SJ		FJ		HR	
	DW	N	DW	N	DW	N
1	0.00471	0.00982	0.00757	0.00869	0.00376	0.00255
2	0.00325	0.00238	0.00844	0.00766	0.00638	0.00319
3	0.00376	0.00330	0.00467	0.00575	0.00376	0.00450
4	0.00349	0.00248	0.00669	0.00252	0.00566	0.00234
5	0.00415	0.00615	0.00668	0.01254	0.00259	0.00431
6	0.00249	0.00610	0.00712	0.00864	0.00454	0.00537
7	0.00595	0.00805	0.00864	0.00810	0.00556	0.00254
8	0.00556	0.00522	0.00629	0.00473	0.00551	0.00459
Average	0.00417	0.00544	0.00701	0.00733	0.00472	0.00367
sd	0.00118	0.00267	0.00126	0.00302	0.00128	0.00116

	Total P (mg/plant)					
	NS		KN		KR	
	DW	N	DW	N	DW	N
1	0.00471	0.00280	0.00491	0.00284	0.00531	0.01295

2	0.00242	0.00238	0.00582	0.00383	0.00376	0.00383
3	0.00309	0.00199	0.00364	0.00376	0.00269	0.00482
4	0.00452	0.00280	0.00424	0.00241	0.00543	0.00344
5	0.00317	0.00307	0.00683	0.00361	0.00917	0.00273
6	0.00434	0.00551	0.00498	0.00493	0.00498	0.00151
7	0.00439	0.00302	0.00278	0.00346	0.00390	0.00454
8	0.00488	0.00302	0.00376	0.00317	0.00439	0.00215
Average	0.00394	0.00308	0.00462	0.00350	0.00495	0.00450
sd	0.00091	0.00105	0.00130	0.00075	0.00193	0.00360
